# Comparative Quantification of Arterial Lipid by Intravascular Photoacoustic-Ultrasound Imaging and Near-Infrared Spectroscopy-Intravascular Ultrasound

**DOI:** 10.1007/s12265-018-9849-2

**Published:** 2018-11-28

**Authors:** Ayeeshik Kole, Yingchun Cao, Jie Hui, Islam A. Bolad, Mouhamad Alloosh, Ji-Xin Cheng, Michael Sturek

**Affiliations:** 1 Department of Cellular & Integrative Physiology, Indiana University School of Medicine, 635 Barnhill Drive, MS 385, Indianapolis, IN 46202 USA; 2 Weldon School of Biomedical Engineering, Purdue University, West Lafayette, IN 47907 USA; 3 Department of Physics and Astronomy, Purdue University, West Lafayette, IN 47907 USA; 4 Roudebush VA Medical Center and Krannert Institute of Cardiology, Indiana University School of Medicine, Indianapolis, IN 46202 USA; 5 Department of Biomedical Engineering, Department of Electrical and Computer Engineering, Photonics Center, Boston University, Boston, MA 02215 USA

**Keywords:** Atherosclerosis, Intravascular imaging, Photoacoustic imaging, Near-infrared spectroscopy, Lipid core plaque, Perivascular adipose tissue, Swine, Human

## Abstract

Intravascular photoacoustic-ultrasound (IVPA-US) imaging and near-infrared spectroscopy-intravascular ultrasound (NIRS-IVUS) are two hybrid modalities that detect arterial lipid, with comparison necessary to understand the relative advantages of each. We performed in vivo and ex vivo IVPA-US imaging of the iliac arteries of Ossabaw swine with metabolic syndrome (MetS) and lean swine to investigate sensitivity for early-stage atherosclerosis. We repeated imaging ex vivo with NIRS-IVUS for comparison to IVPA-US and histology. Both modalities showed significantly greater lipid in MetS vs. lean swine, but only IVPA-US localized the lipid as perivascular. To investigate late-stage atherosclerosis, we performed ex vivo IVPA-US imaging of a human coronary artery with comparison to NIRS-IVUS and histology. Two advanced fibroatheromas were identified, with agreement between IVPA-measured lipid area and NIRS-derived lipid content. As confirmed histologically, IVPA-US has sensitivity to detect lipid content similar to NIRS-IVUS and provides additional depth resolution, enabling quantification and localization of lipid cores within plaques.

**Electronic supplementary material:**

The online version of this article (10.1007/s12265-018-9849-2) contains supplementary material, which is available to authorized users.

## Introduction

Over the past two decades, morphometric data from autopsy specimens have advanced our understanding of atherosclerotic plaque types and their progression to major adverse cardiovascular events [1, 2]. Evidence has shown that thin-capped fibroatheromas (TCFAs) are the plaque type most vulnerable to rupture and are defined by hallmarks of a thin fibrous cap, a large lipid-rich necrotic core, and inflammatory infiltrate [3–5]. Development of intravascular imaging tools to detect vulnerable plaques has been the focus of intense investigation, yet none have been shown to reliably identify TCFAs. Detection of vulnerable plaques will be instrumental in elucidating the mechanisms underlying lesion progression, the development of preventive and therapeutic interventions, and ultimately, reducing CAD-related morbidity and mortality [6–9].

Current intravascular imaging modalities aim to detect hallmarks of TCFAs as a marker of vulnerability. For example, near-infrared spectroscopy (NIRS) uses broadband light absorption to identify lipid-rich plaque and is complemented with intravascular ultrasound (IVUS), which has long been used independently to image vessel and plaque morphology only. The NIRS-IVUS hybrid modality has been successfully validated against histopathology to identify lipid-rich plaques, differentiate between culprit and non-culprit segments, and reasonably predict cardiovascular outcomes in CAD patients [10–14]. Yet, NIRS-IVUS is limited by lack of depth (or radial) resolution, distinguishing only where vascular lipid is circumferentially. This modality cannot provide information about the dimensions of lipid deposition, which is necessary for optimally assessing vulnerability, monitoring lesion progression, and pathophysiological involvement of arterial lipids.

Intravascular photoacoustic (IVPA) imaging has been the latest addition to the list of modalities and uses ultrasound-based detection of optical absorption as a means of contrast. Photoacoustic (PA) imaging relies on the principle that small absorbance of light by C-H bonds abundant in lipids cause thermoelastic expansion and generation of detectable acoustic waves. As a result, IVPA imaging benefits from both optical- and acoustic-based modalities: label-free chemical selectivity and ample penetration depth [15–18]. In IVPA catheters, the optical fiber and ultrasound (US) transducer have been miniaturized to fit and examine overlapping fields of view [19–22]. Thus, IVPA-US operates inherently as a hybrid intravascular catheter, capable of producing complementary cross-sectional images of vascular morphologic information and depth-resolved, lipid-specific compositional information via US and PA channels, respectively [23–25]. IVPA-US imaging has moved towards clinical translation, as limitations such as image acquisition speed and co-registration of channels have been addressed in recent years [20–22, 26, 27]. Yet, there remain significant limitations. For instance, while IVPA-US imaging has been shown to be feasible through blood, optimal sensitivity is still achieved with blood clearance or dilution with saline heavy water (D_2_O) [20, 22].

As both IVPA-US and NIRS-IVUS detect the lipid-rich component contributing to a vulnerable plaque phenotype, comparison between the two hybrid modalities is warranted. Our aim was to elucidate the relative advantages and shortcomings of each modality.

Here, we demonstrate the ability of in vivo and ex vivo IVPA-US imaging to quantify increased perivascular adipose tissue (PVAT), a known mediator of early atherosclerotic plaque formation, using Ossabaw miniature swine, a previously characterized large animal model of metabolic syndrome (MetS) and dyslipidemia [28–31]. We further compare IVPA-US imaging to ex vivo NIRS-IVUS and histopathology. The ability to detect and localize arterial lipids in early atherosclerosis will provide insights into the role of PVAT in lesion progression. This mechanism involves “outside-to-inside” signaling from PVAT-derived adipokines (e.g., leptin) that diffuse to the vascular wall and synergize with more classical “inside-to-outside” mediators (e.g., lipoproteins) to promote atherosclerosis [28–31]. Furthermore, we show comparative quantification and localization of advanced atherosclerotic plaques in a fresh human coronary artery by ex vivo IVPA-US compared to NIRS-IVUS and histopathology. To our knowledge, this is the first comparison of IVPA-US to NIRS-IVUS imaging, and other existing modalities, such as single-modality grayscale IVUS.

## Materials and Methods

### Animal Care and Use

Ossabaw miniature swine (*n* = 3) were maintained to meet the characteristics of MetS for 8 months. For 7 of 8 months, they were fed a hypercaloric, atherogenic diet (1 kg/day) and were approximately age 16 months at euthanasia. For a lean control, a subset of swine (*n* = 2) were fed a standard chow diet (0.7 kg/day). Metabolic data including body weight, blood pressure, serum chemistry, and plasma lipids were collected to confirm development of MetS and dyslipidemia. Development of MetS and preclinical vascular disease in this naturally progressive animal model of human disease has been characterized extensively in previous work [32, 33].

Anesthesia was induced via intramuscular injection of telazol (5–6 mg/kg) and xylazine (2.2 mg/kg), and maintained with 2–4% isoflurane mixed with 100% oxygen.

### IVPA-US Imaging System and Catheter

**Fig. 1:**
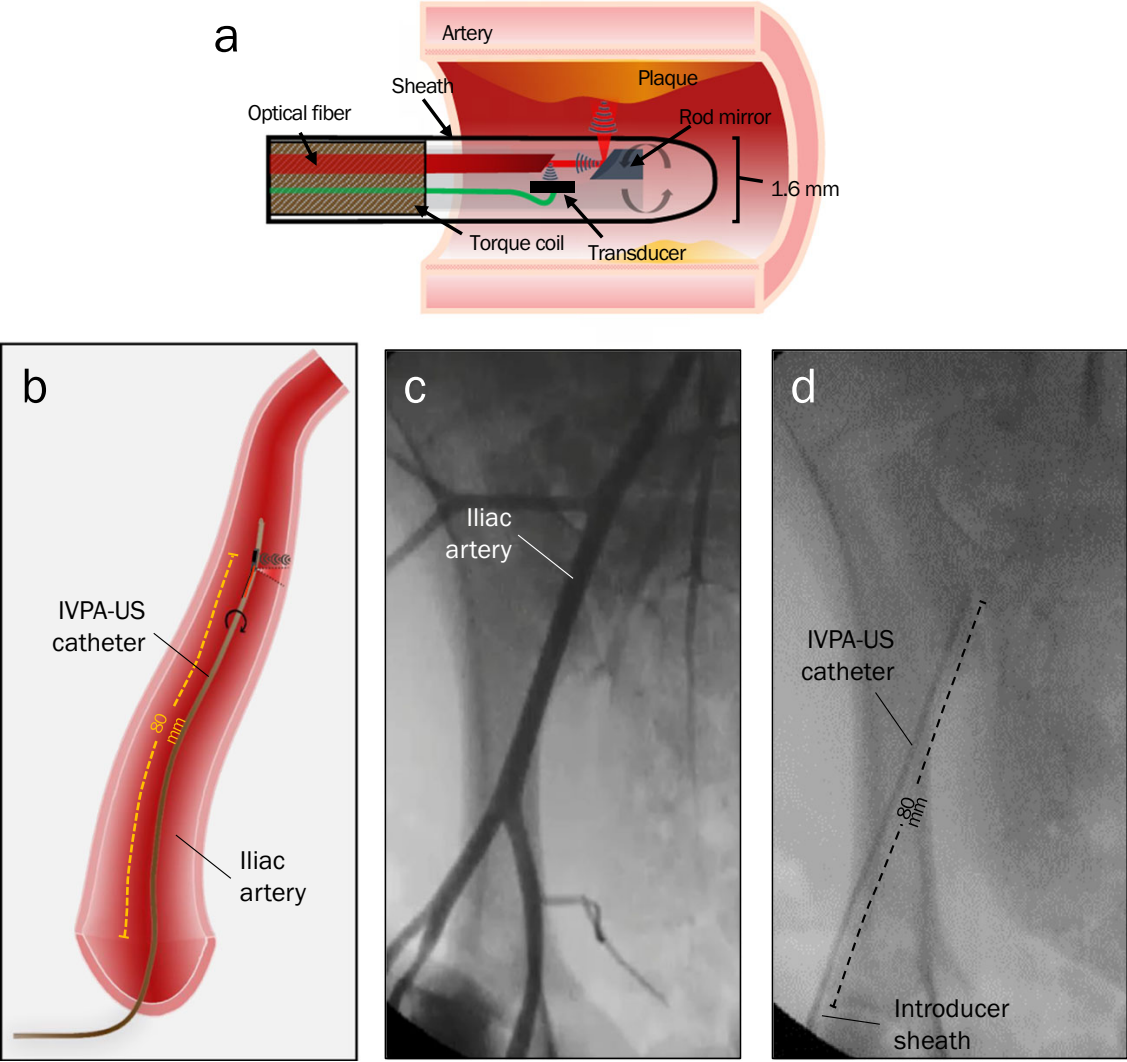
IVPA-US imaging probe and in vivo imaging procedure. **a** Schematic of the IVPA-US collinear catheter design, in which the acoustic and optical paths overlap, after a series of reflections off the rod mirror and optical fiber surfaces. **b** Schematic of the in vivo procedure, in which the IVPA-US catheter was advanced 80 mm into the iliac artery of swine. **c** Angiogram with contrast to visualize vascular anatomy. **d** Angiogram without contrast to confirm IVPA-US catheter placement at 80 mm distal to the introducer sheath

The high-speed IVPA-US imaging system used in this work has been described in detail elsewhere [21, 22, 27]. Briefly, a nanosecond Nd:YAG pumped optical parametric oscillator excitation laser source emitting at 1730 nm with a 2-kHz repetition rate was used for photoacoustic excitation. A collinear catheter with a 42-MHz transducer was used for all swine imaging (Fig. 1a). For human coronary artery imaging, a modified quasi-collinear probe design was used, in which angling of the transducer and reduction of reflection surfaces maintained optical and acoustic co-registration and improved US quality (supplementary Fig. S1) [22]. The laser pulse energy delivered to the tissue was controlled to a fluence of 50 mJ/cm^2^, below the ANSI laser safety standard for of 1 J/cm^2^ at 1730 nm for the skin. The catheter was enclosed within a protective sheath composed of polyurethane and had an outer diameter of 1.6 mm. For all IVPA-US imaging, the catheter sheath was flushed with deuterium oxide (D_2_O) due to decreased optical absorption compared to H_2_O.

### In vivo Imaging: Angiography, IVUS, and IVPA-US

A 7-Fr introducer sheath was inserted in the right femoral artery of swine and heparin was administered (50 U/kg). Next, a 7-Fr guiding catheter was advanced to access the right iliac artery, through which our 4.8-Fr, 42-MHz hybrid IVPA-US catheter was advanced 80 mm distally and two automated pullbacks were recorded at 0.5 mm/s and 8 frames per second (fps) imaging speed (Fig. 1b–d). Next, a 3.2-Fr, 45-MHz single-modality grayscale IVUS catheter (Revolution, Volcano, Corp.) was advanced 80 mm distally and a pullback was recorded at 0.5 mm/s and 30 fps. In one lean control animal, right and left iliac arteries were both imaged. All in vivo imaging was performed through luminal blood, without clearance or dilution. Following imaging, the animals were euthanatized by isoflurane overdose and cardiectomy.

### Ossabaw Swine Tissue Recovery and Preparation

Following euthanasia, the iliac artery was isolated, marked with a suture knot 80 mm distal to the guiding catheter, and dissected out of the animal. Side branches and the distal end were ligated with suture for pressure-perfusion during ex vivo imaging, followed by formalin fixation.

### Human Tissue Recovery and Preparation

A fresh human heart was harvested from a 59-year-old male undergoing transplant surgery, within 24 h of explant. The patient had coronary heart disease risk factors of smoking, hypertension, and obesity. The right coronary artery was excised, the ostium was cannulated with a 6-Fr introducer sheath, and side branches and the distal end of the artery were ligated with suture for pressure-perfusion. Artery anatomy was observed by ex vivo angiography. All imaging was performed within 48 h of dissection.

### Ex vivo Imaging: IVPA-US and NIRS-IVUS

The hybrid IVPA-US catheter was advanced to the 80-mm mark of Ossabaw iliac artery segments and perfused with 1X phosphate buffered saline at approximately 70 mL/min, during which an automated pullback was recorded at 0.25 mm/s and 4fps imaging speed. This procedure was repeated with a 3.2-Fr, 40-MHz hybrid NIRS-IVUS catheter (TVC Insight, Infraredx, Inc.) with a 0.5-mm/s pullback speed and 16 fps imaging speed. Human coronary artery imaging was performed following the same protocol, with the pullbacks starting approximately 70 mm distal to the introducer sheath.

### IVPA-US and NIRS-IVUS Quantitative Analysis

To compare pullbacks from two hybrid modalities collected with different optimal frame rates and pullback speeds, we used angiography and landmarks, such as the introducer sheath and fiduciary side branches, to align frames.

IVPA-US imaging data were post-processed to reduce noise and pixels at which PA intensity was greater than a set threshold were counted per cross-sectional frame to calculate lipid area (four times the background noise for all ex vivo data sets and five times the background noise for all in vivo data sets). Thresholds were determined by an investigator blind to sample group, other imaging results, and histology. For human coronary artery imaging, the lipid core volume was calculated per lesion within a 4-mm segment by identifying depth and angle boundaries within which pixels were counted. All lipid area measurements were calculated using MATLAB software (MathWorks, Inc.).

Cross-sectional lipid areas were binned and averaged for correlation analysis between repeat in vivo and ex vivo pullbacks to assess reproducibility of data.

The NIRS-IVUS TVC Insight system was used to generate two-dimensional “chemograms” representing probability of the presence of lipid by a yellow color, with longitudinal pullback position on the *x*-axis and circumferential position on the *y*-axis. As NIRS-IVUS spectral depth is 1 mm or less, lipid area was estimated by measuring the yellow-colored regions on the 2D chemograms. The system also generated “block chemograms,” representing the artery in 2-mm segments with discrete colorized representation of the probability of lipid present. We defined blocks colored red as having no lipid present (*p* < 0.57) and all other colors as having increased probability of lipid present [34]. The total lipid core burden index (LCBI) and maximum LCBI within a 4-mm segment (maxLCBI_4mm_) were also calculated by the system.

Calcification was measured from the IVUS channel of the hybrid modalities and defined as strong echogenicity with acoustic shadowing. The total length was calculated as continuous frames in which calcification was present and maximum arc was calculated as the largest angle of acoustic shadowing. All analysis of the IVUS channel was performed using ImageJ software (National Institutes of Health).

### Histological Preparation and Analysis

Imaged arteries were pressure-fixed using 10% *w*/*v* formalin and placed in formalin overnight. Contralateral control iliac arteries from Ossabaw swine were not pressure-fixed. Arteries were then grossly sectioned in 3- to 4-mm segments. Next, all segments were paraffin embedded, thin sectioned, and stained with hematoxylin and eosin (H&E) and Verhoeff-Van Gieson elastin stain. In the human coronary artery with advanced atherosclerosis, Russel-Movat’s pentachrome stain was used to identify and characterize lipid core plaques [1, 10]. Lipid-rich necrotic cores were defined as morphologically distinct empty and/or clear, granular, mostly anucleate spaces, defined as the light-microscopic characteristics of lipid gruel, with macrophage infiltration, cholesterol clefts, or calcification [1, 2, 35]. Necrotic core area was calculated using ImageJ software.

### Statistics

All data are described as mean ± SEM. Statistical significance was set a priori at *p* < 0.05. Unpaired, two-tailed Student’s *t* tests, Fisher’s exact test, and Pearson’s correlation were used as appropriate using GraphPad Prism statistical software (GraphPad Software, Inc.).

## Results

### IVPA-US Sensitivity for Early-Stage Atherosclerosis in Ossabaw Swine

**Fig. 2:**
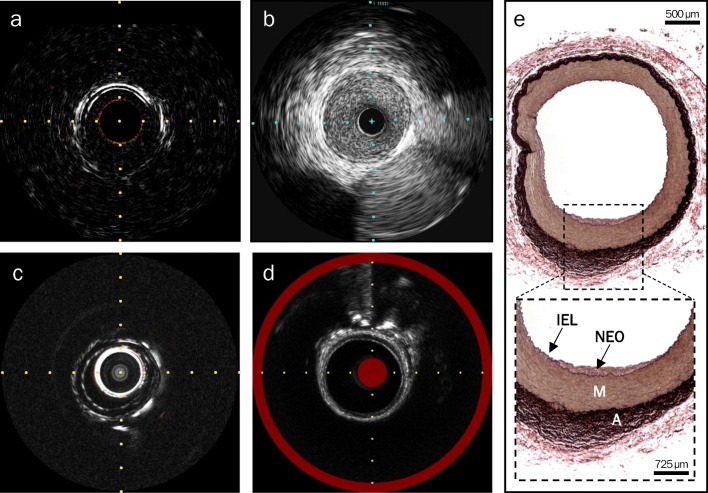
Representative imaging and histology from a MetS Ossabaw swine iliac artery showing no intimal lipid deposition and only early-stage neointimal thickening. **a** In vivo IVPA-US still frame, in which no lipid deposition (absence of red) and mild neointimal hyperplasia (three-layer appearance, grayscale IVUS channel) was observed. **b** In vivo single-modality grayscale IVUS still frame, depicting the three-layer appearance. **c** Ex vivo IVPA-US still frame, in which no lipid deposition and the three-layer appearance were observed. **d** Ex vivo NIRS-IVUS still frame, in which no lipid deposition (absence of yellow circumferentially) and the three-layer appearance (grayscale IVUS channel) were observed. **e** Verhoeff–Van Gieson stained histological section, showing typical artery morphology with a small area of neointimal thickening (NEO) on the luminal side of the internal elastic lamina (IEL). M media, A adventitia. Horizontal and vertical axis tracings are 1 mm apart

Ossabaw swine on hypercaloric, atherogenic diet developed the characteristics of MetS, including significantly greater total cholesterol, triglycerides, and weight at sacrifice compared to swine in the lean control group (Table 1). Despite the development of MetS in swine, on in vivo and ex vivo iliac artery imaging of both groups, no significant intimal lipid deposition was detected on all modalities, consistent with histology which showed mild vascular disease evident as minimal neointimal hyperplasia (Fig. 2).

**Table 1:** Metabolic characteristics of Ossabaw swine

	Lean (*n* = 2)	MetS (*n* = 3)	*p* value
Sex (M/F)	1/1	0/3	N/A
Weight at sacrifice (kg)	50.5 ± 10.5	95.0 ± 1.5*	0.01
Mean weight gain (kg)	31.0	58.7 ± 3.0	N/A
Plasmalipids			
Cholesterol (mg/dL)	71 ± 12	465 ± 53*	0.01
Triglycerides (mg/dL)	26 ± 2	63 ± 4*	0.01

*p < 0.05

**Fig. 3:**
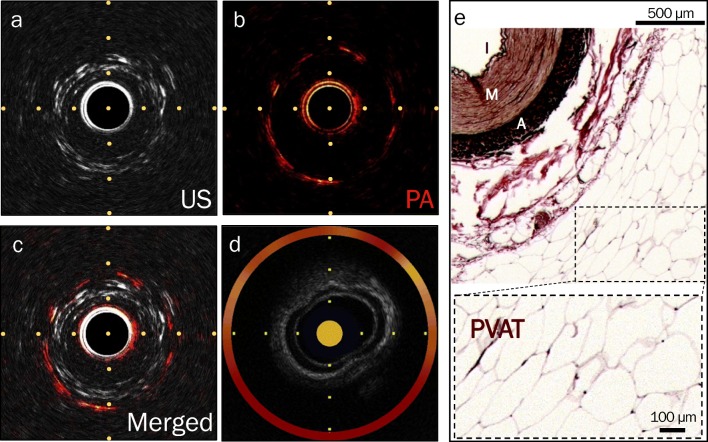
Representative in vivo IVPA-US imaging from a MetS Ossabaw swine iliac artery showing a lipid signal by both modalities. **a** Unmerged IVUS channel still frame showing a three-layer appearance of the iliac artery. **b** Unmerged IVPA channel still frame showing circumferential distribution and depth of lipid signal (red). **c** Merged composite showing depth-resolved lipid signal localized to the perivascular region. **d** NIRS-IVUS still frame showing grayscale IVUS channel and lipid signal (yellow) circumferentially, but not the precise radial depth into the arterial wall and perivascular regions. **e** Verhoeff–Van Gieson stained histological section showing abundant perivascular adipose tissue (PVAT, inset), noted by the presence of round and empty adipocytes. I intima, M media, A adventitia. Horizontal and vertical axis tracings are 1 mm apart

However, in specific segments of the iliac arteries, low levels of lipid signal were detected by both IVPA-IVUS and NIRS-IVUS, which were further localized to the perivascular region and confirmed as PVAT by histology (Fig. 3). Similar signal was detected by NIRS-IVUS at corresponding regions of interest, albeit without localization of the lipid depot radially due to lack of depth resolution (Fig. 3d).

**Fig. 4:**
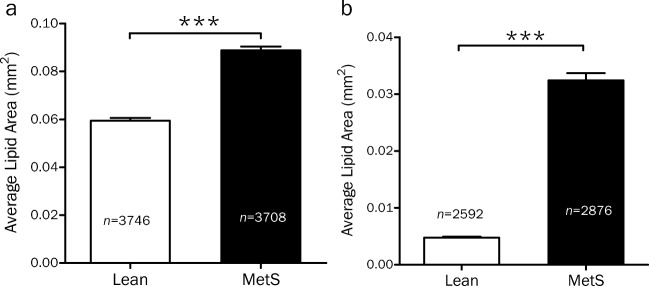
Average cross-sectional lipid area in iliac arteries as determined by IVPA-US imaging. The average cross-sectional lipid area, as calculated by photoacoustic signal intensity per frame above a pre-determined threshold, is significantly greater in the iliac arteries of swine with MetS as compared to lean swine, both by in vivo (**a**) and ex vivo (**b**) imaging; *** *p* < 0.0001; *n* refers to number of cross-sectional frames analyzed

On in vivo IVPA imaging, we found that the average cross-sectional lipid area was significantly greater in the iliac arteries of swine with MetS as compared to lean swine (0.089 ± 0.016 mm^2^ vs. 0.059 ± 0.001 mm^2^; *p* < 0.0001) (Fig. 4a). These results were also observed on ex vivo imaging (0.032 ± 0.001 mm^2^ vs. 0.005 ± 0.0002 mm^2^; *p* < 0.0001) (Fig. 4b).

### Correlation of IVPA-US with NIRS-IVUS in Early-Stage Atherosclerosis

On ex vivo NIRS-IVUS imaging, we found similar results to IVPA-US imaging, as arteries of swine with MetS had significantly more 2-mm block chemograms with increased probability of lipid present than arteries of lean swine (8/117 blocks with lipid vs. 0/80 blocks with lipid; *p* = 0.02). Taken together, these results conclude IVPA-US is comparable to NIRS-IVUS in sensitivity for the detection of increased lipid content. Furthermore, depth resolution of IVPA-US imaging allowed localization of lipid deposition to PVAT, a known mediator of early atherogenesis [28–31].

### IVPA-US Imaging In vivo Safety and Reproducibility

We evaluated the safety of the in vivo imaging procedure and reproducibility of our results. On angiography, we did not observe significant vasospasm after IVPA-US pullback (supplementary Fig. S2a-b). On histology, we did not observe any significant damage to the internal elastic lamina of imaged arteries compared to contralateral control arteries (supplementary Fig. S2c-f). Additionally, in three swine iliac arteries, we performed repeat in vivo pullbacks and there was significant correlation of lipid area between pullbacks (*p* < 0.0001 for all data sets) (supplementary Fig. S3). In a MetS-group swine iliac artery, we further compared lipid area measures from in vivo pullbacks with ex vivo pullback data and we also found significant correlation (*p* < 0.05 for all data sets) (supplementary Fig. S4).

### IVPA-US Sensitivity for Late-Stage Atherosclerosis in a Human Coronary Artery

**Fig. 5:**
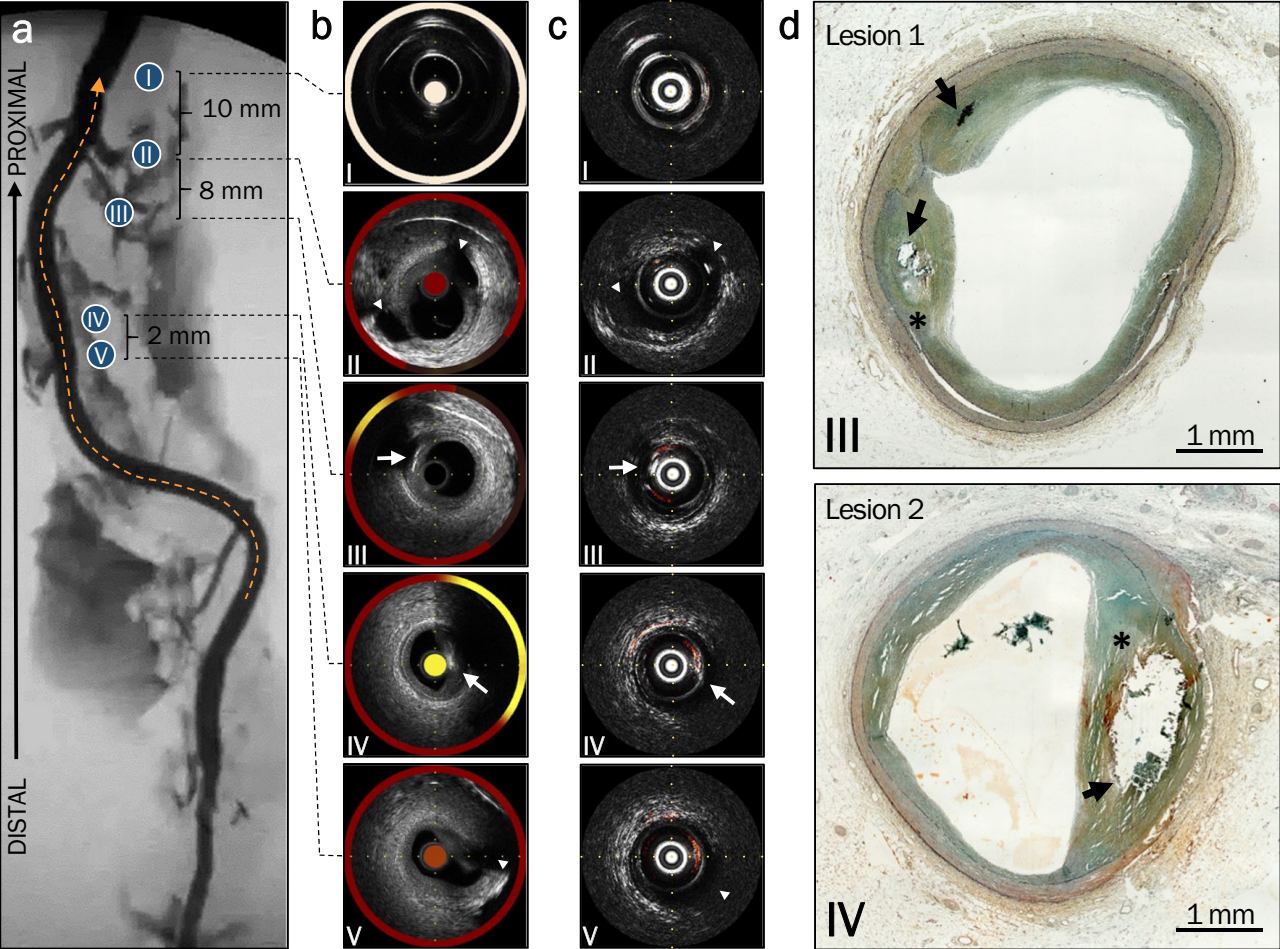
Correlation between modalities in a fresh human coronary artery. **a** Ex vivo angiogram of the artery, in which the sheath (I), side branches (II, V), and lesions locations (III, IV) are identified. Location III refers to Lesion 1 and location IV to Lesion 2. **b**, **c** Corresponding cross-sectional still frames are shown from NIRS-IVUS (**b**) in which lipid is shown with yellow and from IVPA-US (**c**) in which lipid is represented by red. Notable side branches are indicated by arrowheads and calcification by arrows. **d** Movat’s pentachrome stained histological sections of lipid core lesions (*) with calcification (arrows). Horizontal and vertical axis tracings are 1 mm apart

On ex vivo imaging of a fresh human right coronary with atherosclerosis, we identified two advanced fibroatheromas with calcification on both IVPA-US and NIRS-IVUS (Fig. 5). We defined the atheroma proximal to the introducer sheath as lesion 1 and the distal atheroma as lesion 2 (Table 2). On both hybrid modalities, calcification was evident on ultrasound by strong echogenicity with acoustic shadowing (Fig. 5b, c, panels III and IV). Furthermore, lipid was present as indicated by yellow color on the circumferential chemogram by NIRS-IVUS and red color on cross-sectional images by IVPA-US. Characterization of the atherosclerotic features in both lesions were confirmed by histopathology (Fig. 3d).

**Table 2:** Comparison of lesion characteristics from ex vivo imaging of a human right coronary artery by NIRS-IVUS and IVPA-US

	Lesion 1 (proximal)	Lesion 2 (distal)
NIRS-IVUS measured lesion characteristics		
maxLCBI_4mm_	N/A	326
Total lipid area (mm^2^)	5.22	26.38
Total calcified length (mm)	3.1	2.0
Maximum arc of calcification (^o^)	46.2	66.7
IVPA-US measured lesion characteristics		
Average lipid area_4mm_ (mm^2^)	0.15 ± 0.004	0.40 ± 0.03
Total lipid area_4mm_ (mm^2^)	9.57	25.95
Lesion lipid core volume (mm^3^)	0.11	0.25
Total calcified length (mm)	3.4	2.4
Maximum arc of calcification (^o^)	58.1	66.4

On IVPA-US imaging, we calculated the average cross-sectional lipid area within a 4-mm segment for both lesions. Lesion 1 had an average lipid area of 0.15 ± 0.004 mm^2^ (range 0.08–0.24) and lesion 2 had an average lipid area of 0.40 ± 0.03 mm^2^ (range 0.04–1.04). Particularly, lesion 2, within the measured 4-mm segment, had a significantly greater average lipid area than the remaining length of artery (0.40 mm^2^ vs. 0.26 mm^2^; *p* < 0.0001). We further measured the total lipid area within a 4-mm segment for each lesion (lesion 1 9.57 mm^2^, lesion 2 25.95 mm^2^).

Lastly, depth resolution and co-registered imaging allowed us to identify the contiguous lipid areas adjacent to calcification as the plaque lipid core and calculated the volumes as 0.11 mm^3^ and 0.25 mm^3^ for lesions 1 and 2, respectively. Relation of the plaque lipid core to calcification and the lumen border is further illustrated by three-dimensional reconstruction of the 4-mm segments (supplementary Videos 1 and 2).

### Correlation of IVPA-US with NIRS-IVUS and Histology in Late-Stage Atherosclerosis

Quantifications by IVPA-US imaging were in agreement with similar measures made by NIRS-IVUS imaging and histopathology. On NIRS-IVUS, we found lesion 2 to have a high maxLCBI_4mm_ value of 326, consistent with the large lipid area and core volume measured by IVPA-US. No maxLCBI_4mm_ was calculated for lesion 1 due to lack of sufficient spectrally valid pixels. Using the generated chemogram, we approximated lipid areas as 5.22 mm^2^ for lesion 1 and 26.38 mm^2^ for lesions 2, comparable to the values measured by IVPA-US.

In addition, we characterized the calcification in both lesions by measuring the total length and maximal arc of calcification, as shown in Table 2. Both IVPA-US and NIRS-IVUS systems showed similar values, demonstrating comparable resolution of the US channel for the identification of calcification.

Lastly, on histopathology we measured histologic necrotic core areas of 0.42 mm^2^ and 1.36 mm^2^ for lesions 1 and 2, respectively. The larger necrotic core area on histopathology compared to the average cross-sectional lipid area by IVPA-US was anticipated, as necrotic cores contained features such as prominent calcification, in addition to lipid.

Comparative imaging by two hybrid modalities and gold-standard histopathology confirm that depth-resolved IVPA-US has the capability to quantify and localize plaque lipid cores with calcification.

## Discussion

Previous studies using NIRS-IVUS clearly show the clinical value of identification of arterial lipid as a prognostic indicator of cardiovascular outcomes [12–14]. Those preliminary studies support the notion that accurate detection of arterial lipid content by a reliable intravascular imaging modality, or combination of multiple modalities, could be predictive of lesions at high risk to progress to cardiovascular events. Multiple prospective studies, including PROSPECT II ABSORB sub-study (NCT02171065) and the Lipid-Rich Plaque Study (NCT02033694), aim to answer this important question [36]. As the only FDA-approved device for measuring lipid-rich plaques, NIRS-IVUS undoubtedly has great promise in clinical practice. Yet, lack of depth resolution limits quantitative imaging of lipid and relies upon LCBI as a derived measure to distinguish between high-risk and low-risk plaques. In other words, NIRS-IVUS provides an excellent biochemical assay of bulk lipid content in sections of artery, but the precise location of lipid in the vascular wall and perivascular compartment is not provided.

Here, we conducted the first head-to-head comparison of IVPA-US to NIRS-IVUS and highlight the value of depth resolution in lipid detection. We have demonstrated the capability of IVPA-US to detect both superficial and deep lipid, i.e., lipid within an advanced plaque core and perivascular lipid of early-stage atherosclerosis.

By ex vivo imaging of a human coronary artery, we identified advanced lipid core plaques by both NIRS-IVUS and IVPA-US. We were able to quantify and localize the lipid at corresponding lesions using IVPA-US only, by virtue of depth resolution with co-registration of the two modalities. A demonstrated advantage with depth-resolved lipid imaging is volumetric quantification of lipid plaque cores, which would permit more accurate longitudinal studies monitoring plaque progression and regression. Alterations in plaque morphology and composition could be more meaningful with visualization of lipid core dimensions and relation to other plaque components (i.e., fibrous cap, calcification). This would aid in elucidation of the prevalence and mechanisms of plaque development, stability, and rupture [37].

Additionally, by in vivo IVPA-US imaging of iliac arteries, we detected early-stage atherosclerotic changes in the form of increased PVAT in Ossabaw swine with MetS versus lean swine. Intravascular catheter detection of PVAT, although not considered vulnerable pathology, would be a valuable measure to investigate its role in early atherogenesis. Many groups have observed an enhanced pro-inflammatory milieu in adipose tissue of patients with CAD versus those without disease, supporting the hypothesis that PVAT exerts paracrine stimulation of atherogenesis in an “outside-to-inside” manner [28, 38]. In cross-sectional clinical studies, PVAT quantity is strongly associated with several atherosclerotic measures, including plaque presence, percent coronary artery stenosis, coronary artery calcium score, and future major adverse cardiovascular events [29, 30]. Lastly, surgical resection of local coronary PVAT in swine models has been shown to attenuate underlying coronary atherosclerosis, providing the first causal evidence [31]. However, PVAT is traditionally measured by computed tomography along the cardiac short axis and co-localization to intravascular measures such as plaque burden are not possible, limiting the ability to assess the causal relationship. In other words, the field currently does not have the tools necessary to answer the question: do local depots of PVAT precede the development of underlying atherosclerosis? This shortcoming can be addressed by IVPA-US, which has penetration depths of up to 6 mm and can image plaque burden and PVAT in one procedure, enabling needed longitudinal natural history studies [21].

Lastly, clinical translation of IVPA-US will require validation against a robust data set of human histopathology conducted ex vivo [10]. Here, we have shown evidence in swine demonstrating correlation between ex vivo imaging through saline and in vivo imaging through blood. Additionally, we have confirmed safety of our in vivo procedure, in which there were no peri-procedural complications. Lastly, we observed no irritation to the artery as indicated by insignificant post-pullback vasospasm and no disruption to the internal elastic lamina.

This study is limited by the lack of intimal lipid in the iliac arteries of Ossabaw swine and subsequent absence of analysis for correlation of in vivo neointimal lipid imaging to histopathology. The observation of increased PVAT depots, no neointimal lipid, and mild neointimal thickening is very intriguing and could provide new insights about atherogenesis. In future studies, we intend to employ longer duration MetS phenotypic development and longitudinal IVPA-US imaging to determine whether increased PVAT precedes an increase in neointimal lipid. We will also address catheter size and flexibility limitations to image coronary arteries, in which atherosclerotic disease develops more predictably [32].

Lastly, our human artery sample size is small and does not include a wide range of atherosclerotic plaque phenotypes. Thus, we were unable to calibrate our thresholding algorithm for IVPA-derived lipid area to correlate with histopathological lipid-rich necrotic core area. We intend to build a larger calibration data set to address this limitation. In addition, we anticipate future application of spectral algorithms to differentiate between cholesterol subtypes, as a surrogate marker of inflammation [39].

## Conclusion

As intravascular imaging modalities for lipid imaging advance in capability and combination with others, comparison between systems will be instrumental in determining the relative advantages and disadvantages of each. We have demonstrated detection of increased lipid deposition in Ossabaw swine with early-stage atherosclerosis versus lean swine by IVPA-US imaging, in agreement with NIRS-IVUS imaging of the same specimens. Further, we have shown detection of late-stage atherosclerotic plaques in human coronary arteries by both modalities. In conducting this comparison, we reveal the advantage of depth-resolved IVPA-US imaging in varying stages of atherosclerosis for the localization and quantification of both superficial and deep lipid content, even extending into the perivascular region.

## Electronic Supplementary Material

ESM 1 (MP4 5854 kb)

ESM 2 (MP4 7241 kb)

ESM 3 (PDF 937 kb)

## Abbreviations

CAD: Coronary artery disease
MetS: Metabolic syndrome
H&E: Hematoxylin and eosin
NIRS: Near-infrared spectroscopy
IVPA-US: Intravascular photoacoustic-ultrasound
PA: Photoacoustic
IVUS: Intravascular ultrasound
PVAT: Perivascular adipose tissue
LCBI: Lipid core burden index
TCFA: Thin-capped fibroatheroma
maxLCBI_4mm_: Maximum lipid core burden within a 4 mm segment
US: Ultrasound
