# Supplementary material for: Comparative Quantification of Arterial Lipid by Intravascular Photoacoustic-Ultrasound Imaging and Near-Infrared Spectroscopy-Intravascular Ultrasound
Source: J Cardiovasc Transl Res. 2018 Nov 28;12(3):211–20. doi: 10.1007/s12265-018-9849-2 (PMC6611754; doi:10.1007/s12265-018-9849-2)
Supplement: Supplementary file 3 — (PDF 937 kb) [file 12265_2018_9849_MOESM3_ESM.pdf]

SUPPLEMENTARY MATERIAL

Comparative quantification of arterial lipid by intravascular photoacoustic-ultrasound imaging and near-infrared spectroscopy-intravascular ultrasound

Ayeeshik Kole, PhD[a,b,1], Yingchun Cao, PhD[b,1], Jie Hui, PhD[c], Islam A. Bolad, MD[d], Mouhamad Alloosh, MD[a], Ji-Xin Cheng, PhD[b,e,], Michael Sturek, PhD[a,b,*]

Author Affiliations:

[a]Department of Cellular & Integrative Physiology, Indiana University School of Medicine, Indianapolis, IN 46202, USA
[b]Weldon School of Biomedical Engineering, Purdue University, West Lafayette, IN 47907, USA
[c]Department of Physics and Astronomy, Purdue University, West Lafayette, IN 47907, USA
[d]Roudebush VA Medical Center and Krannert Institute of Cardiology, Indiana University School of Medicine, Indianapolis, IN 46202, USA
[e]Department of Biomedical Engineering, Department of Electrical and Computer Engineering, Photonics Center, Boston University, Boston, MA 02215, USA

[1]These authors contributed equally to this work.

* Corresponding author at:
Indiana University School of Medicine, 635 Barnhill Drive, MS 385, Indianapolis, IN 46202, USA
E-mail address: msturek@iu.edu
Phone: (317) 274-7772

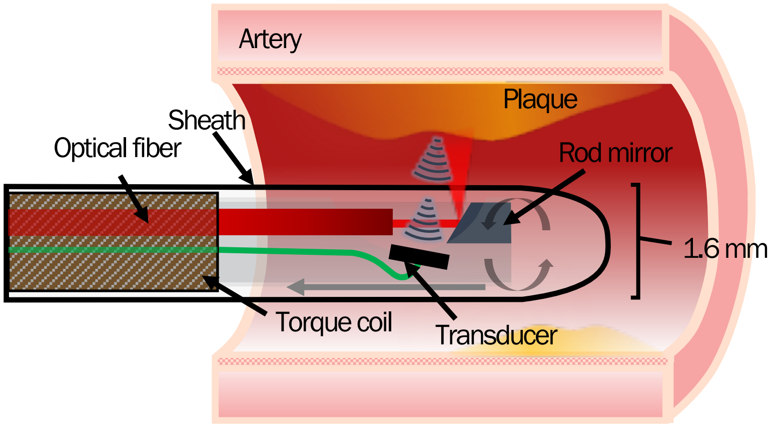

Fig. S1 IVPA-US imaging probe. Schematic of the IVPA-US quasi-collinear catheter design, in which there is sufficient overlap of acoustic and optical paths from a theoretical range of 0.6 mm to 6 mm without loss of ultrasound resolution from multiple reflection points. This design was used for all human artery imaging.

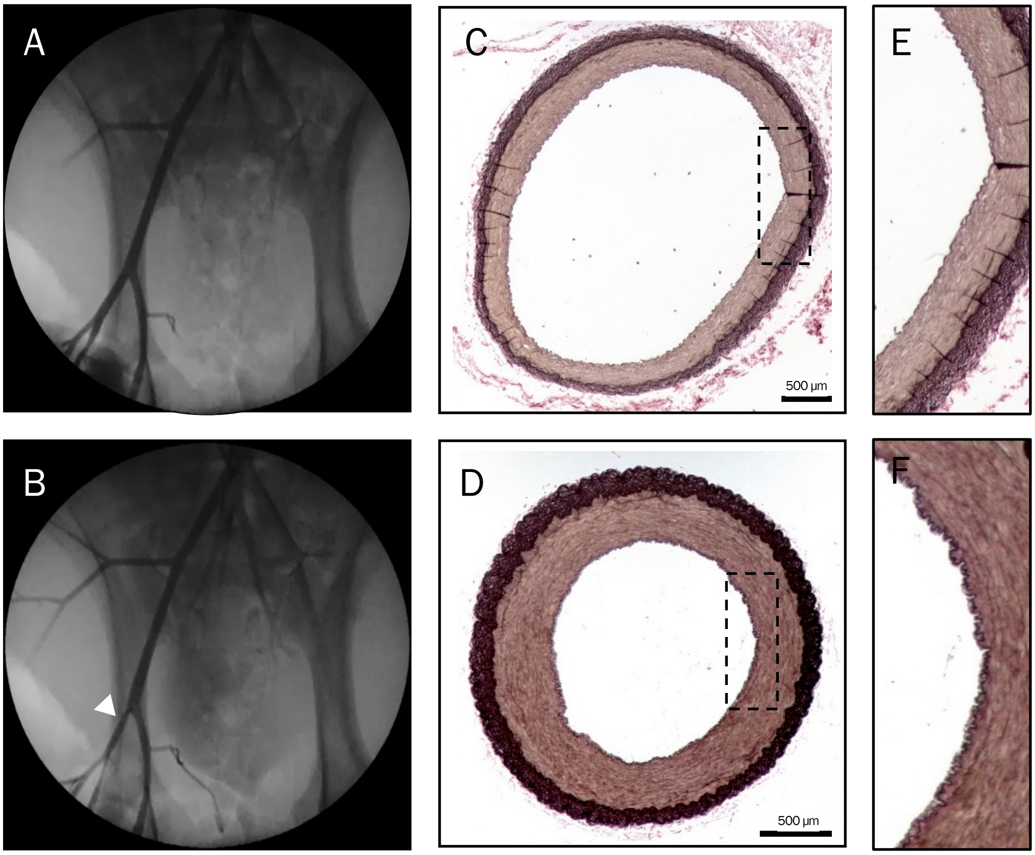

Fig. S2 Sheath performance as measured by post-catheterization vasospasm and endothelial denudation. **a** Pre-IVPA-US angiogram of an artery without catheter intervention. **b** Post-IVPA-US angiogram taken immediately after pullback, showing only mild spasm of the vessel, proximally and adjacent to the introducer sheath (arrowhead). **c** Verhoeff–Van Gieson stained histological section from an imaged iliac artery, showing an intact internal elastic lamina (inset, **e**). **d,f** Uncatheterized contralateral iliac artery as control.

Fig. S3 Reproducibility of *in vivo* procedure and results. In three swine, we repeated our pullback procedure to study the reproducibility of cross-sectional lipid area measurements (mm^2) along the length of pullbacks. Paired pullback 1 and 2 lipid areas are plotted on *x*- and *y*-axes, respectively. In each animal, we observed significant correlation between the initial (Pullback 1) and repeat (Pullback 2) data sets. All data sets were binned and averaged equally to 50 data points representing the total pullback length.

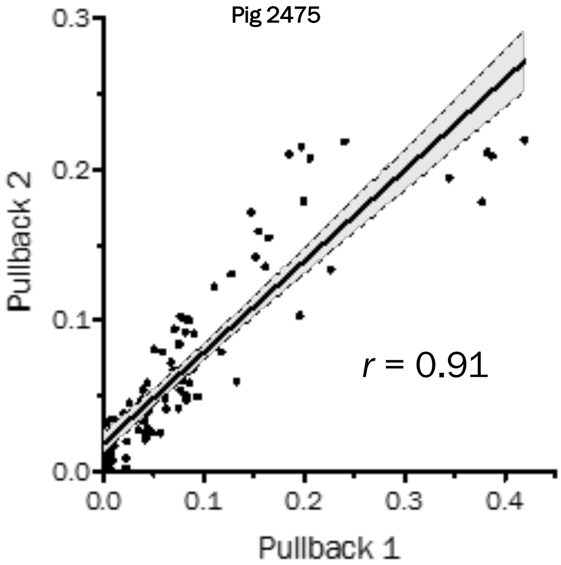

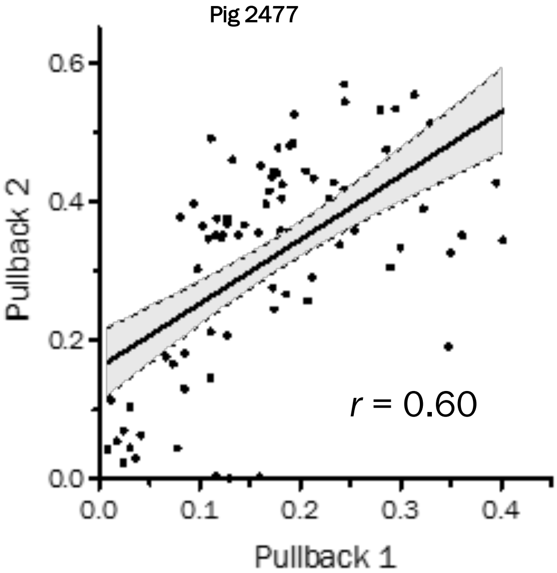

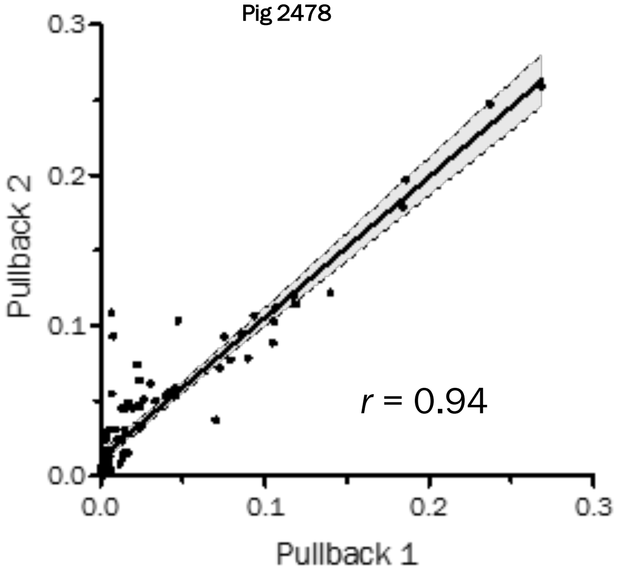

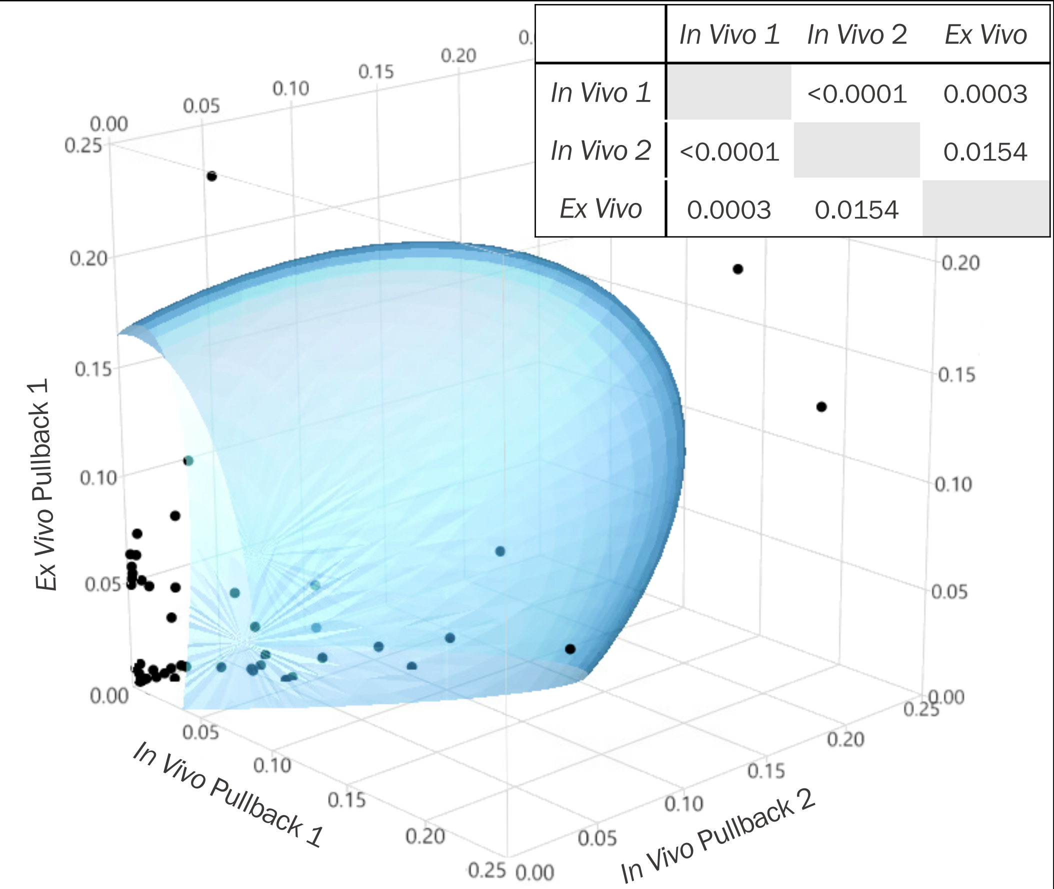

	In Vivo 1	In Vivo 2	Ex Vivo
In Vivo 1		<0.0001	0.0003
In Vivo 2	<0.0001		0.0154
Ex Vivo	0.0003	0.0154	

Fig. S4 Correlation plot between *in vivo* and *ex vivo* results with 95% confidence ellipsoid. In a swine with MetS, in which we had minimal noise in two repeat *in vivo* pullbacks (8 fps imaging speed and 0.5 mm/s pullback speed) and one *ex vivo* pullback (4 fps imaging speed and 0.25 mm/s pullback speed), we found cross-sectional lipid areas (mm^2, plotted on *x,y,z*-axes) to significantly correlate between all data sets (all $p < 0.05$). All data sets were binned and averaged equally to 50 data points representing the total pullback length.
